# A transformer-based embedding approach to developing short-form psychological measures

**DOI:** 10.3389/fpsyg.2025.1640864

**Published:** 2025-08-13

**Authors:** Se-Jin Jung, Jang-Won Seo

**Affiliations:** Department of Psychology, Jeonbuk National University, Jeonju, Republic of Korea

**Keywords:** short-form development, item reduction, transformer-based embedding, semantic clustering, psychological measures

## Abstract

**Introduction:**

Developing short-form psychological measures is essential for reducing respondent burden, saving time, and conserving resources. However, existing short-form development approaches typically require full-scale administration and rely on factor analysis or machine learning techniques based on response data.

**Methods:**

This study proposes a novel, data-independent method for item reduction using transformer-based semantic embeddings. Items from the International Personality Item Pool Big-Five Factor Markers (IPIP-50) were embedded using the sentence-t5-xxl model to generate dense semantic representations. These embeddings were clustered via K-means, and representative items were selected based on their proximity to cluster centroids.

**Results:**

The resulting 30-item short form preserved the original five-factor structure and demonstrated strong psychometric properties. When compared with Classical Test Theory and a Genetic Algorithm, the proposed method achieved comparable levels of reliability, convergent validity, and predictive performance.

**Discussion:**

These findings highlight the potential of transformer-based embedding approaches for efficient item reduction and item development. The results support the feasibility of a resource-efficient, linguistically grounded alternative to data-dependent reduction methods.

## 1 Introduction

In self-report psychological assessments, an excessive number of items can increase respondent fatigue, leading to inattentive or random responses, which may compromise the reliability and validity of the results (Herzog and Bachman, 1981). Furthermore, longer instruments require more administration time, posing practical limitations. Conversely, reducing the number of items without adequately representing the construct's structural dimensions may also result in decreased reliability and validity (Smith et al., 2000). Therefore, developing short forms that maintain both reliability and validity remains a critical objective in psychological measurement.

For these reasons, there has been sustained interest in reducing the number of items in psychological assessments without compromising their psychometric quality. One traditional approach is Classical Test Theory (CTT), which selects items based on their correlations with total scores and evaluates internal consistency using Cronbach's alpha (Lord and Novick, 2008).

While CTT offers advantages such as computational simplicity and ease of interpretation, it also has notable limitations. The method may retain redundant items due to high inter-item correlations and, because it is based on univariate analysis, may fail to capture the multidimensional nature of psychological constructs (Sijtsma, 2009).

Principal Component Analysis (PCA) has also been used as a traditional statistical method for item reduction. PCA identifies orthogonal linear combinations of observed variables that account for the maximum variance in the data (Jolliffe, 2002). Items with high loadings on the first few principal components are often retained to construct a reduced form. However, like CTT, PCA is heavily influenced by inter-item correlations. This reliance can lead to the removal of theoretically important items simply due to statistical redundancy, potentially compromising content validity (Kriegel et al., 2008; Zheng et al., 2020).

To address these limitations, Item Response Theory (IRT) has been proposed as a more refined method for item selection and scale construction. Compared to CTT, IRT offers several advantages, including the ability to estimate item parameters independently of the sample, provide item-level information, and model measurement precision across the latent trait continuum (Hambleton et al., 1991). These properties allow for more flexible and detailed assessments, especially in the development of adaptive or shortened forms.

However, IRT does not systematically explore all possible item combinations or automate the search for the most optimal item sets, leaving room for subjective decisions by researchers during the item reduction process (Yarkoni, 2010).

To overcome these limitations, recent studies have explored machine learning–based approaches for item reduction. For example, Yarkoni (2010) applied a machine learning technique known as the Genetic Algorithm (GA) to select and reduce questionnaire items. GA is an optimization algorithm inspired by biological evolution, beginning with a randomly generated population and iteratively improving solutions across generations (Holland, 1992). This method is particularly well suited to identifying optimal solutions for complex problems (Goldberg et al., 1989). Using GA, Yarkoni successfully shortened existing questionnaires while maintaining internal consistency, thereby demonstrating the potential of machine learning techniques for item reduction in psychological assessment.

However, a major limitation of both traditional and modern item reduction methods is their reliance on response data, which necessitates prior administration of the full questionnaire. Additionally, methods such as the Genetic Algorithm (GA) are probabilistic in nature, meaning that their outcomes can vary across different runs, even under the same conditions (Katoch et al., 2021). With the exception of manual selection based on expert judgment, very few approaches allow for item reduction without first administering the questionnaire (Howard, 2018). In response to this limitation, the present study aimed to develop an objective method for item reduction that does not require prior data collection or pilot testing.

Recent advances in artificial intelligence have led to increased interest in Large Language Models (LLMs). LLMs are artificial intelligence systems trained on large-scale text data to understand and generate human-like language (Chang et al., 2024). To generate meaningful language, these models must first comprehend input text, which involves converting language into a numerical form—a process known as embedding.

Embedding refers to the transformation of linguistic information into numerical vector representations. Through this process, computers can computationally process semantic relationships between words, contextual dependencies, and latent meaning structures. A key property of embedding is that words or texts with similar meanings are represented by vectors that are closer in the embedding space (Rodriguez and Spirling, 2022). By leveraging these semantic distances between vectors, LLMs can effectively interpret the linguistic meaning of text (Rodriguez and Spirling, 2022).

In recent scale development research, there has been growing interest in large language model (LLM)-based embedding approaches that explore the semantic structure of scale items. Within this emerging trend, studies have applied LLM embeddings such as BERT and SBERT combined with cosine similarity to quantify semantic consistency, reduce semantic redundancy while recovering factor structures, or reliably infer item correlation patterns (Hernandez and Nie, 2023; Guenole et al., 2024; Hommel and Arslan, 2024).

In the present study, we applied transformer-based embedding techniques to reduce questionnaire items without relying on prior response data. By numerically encoding the semantic content of each item and clustering them based on vector proximity, we aimed to generate a data-independent short form.

To evaluate this approach, we applied the method to a widely used personality assessment, the International Personality Item Pool 50-item Big-Five Factor Markers (IPIP-50; Goldberg et al., 2006). We then tested the validity of the reduced items, their semantic correspondence with the original items, and the method's effectiveness in comparison to other established item reduction techniques.

## 2 Methods

### 2.1 Samples

Data were obtained from an online administration of the International Personality Item Pool 50-item Big-Five Factor Markers (IPIP-50), which is publicly available through the Open-Source Psychometrics Project (https://openpsychometrics.org/tests/IPIP-BFFM/). Developed based on the publicly available IPIP database, the scale was designed to be freely accessible and is widely used on online platforms. A total of 1,013,558 individual responses were collected.

### 2.2 Measures

#### 2.2.1 International Personality Item Pool 50-item Big-Five Factor Markers (IPIP-50)

The IPIP-50 is a self-report personality assessment designed to measure the Big Five personality traits (Goldberg et al., 2006). Grounded in the Big Five theory, the IPIP-50 assesses personality across five dimensions: Extraversion, Agreeableness, Conscientiousness, Emotional Stability, and Openness to Experience. The scale consists of 50 items, with 10 items corresponding to each of the five traits. Items are rated on a 5-point Likert scale indicating the extent to which the statement applies to the respondent.

### 2.3 Data preprocessing

First, variables irrelevant to the present study (e.g., response time per item) were excluded. Next, cases with missing item responses were identified and removed, resulting in the exclusion of 140,907 responses. To further ensure data quality, Mahalanobis distance was applied to detect multivariate outliers (Kline, 2004), and 61,208 responses were excluded based on a significance threshold of *p* < 0.01. As a result of these preprocessing steps, a total of 813,226 valid responses remained for analysis.

In preparation for semantic embedding and clustering, reverse-scored items were rephrased into their positively worded counterparts (e.g., “I don't talk a lot” was reworded as “I talk a lot”), and the corresponding response scores were adjusted accordingly to reflect direct scoring.

### 2.4 Item reduction

To compare the proposed method with existing item reduction techniques, four approaches were applied to shorten the 50-item IPIP scale to 30 items: (1) a traditional method based on CTT, (2) a machine learning-based method using GA, (3) a factor analytic method using Principal Component Analysis (PCA), and (4) the transformer embedding-based method (TE) developed in the present study.

#### 2.4.1 CTT-based item reduction

In accordance with the principles of CTT, items were reduced by calculating the correlation between each item and the total score of its corresponding subscale. Items within each of the five factors were then ranked in descending order based on these correlations. The top six items from each factor were selected, resulting in a 30-item shortened version of the IPIP-50.

#### 2.4.2 GA-based item reduction

The GA-based item reduction method was implemented using Python and R, based on the approach introduced by Yarkoni (2010). First, the Graded Response Model (GRM; Samejima, 1969) was used to estimate item discrimination and threshold parameters, using the R package “mirt”. Second, a genetic algorithm was applied using the Distributed Evolutionary Algorithms in Python (DEAP) library. The fitness function was defined as the sum of four components: (a) the correlation between the reduced-form and full-scale total scores, (b) internal consistency measured by Cronbach's alpha, (c) average item information at θ = 0, and (d) average item discrimination. which served as a basis for evaluating each item's contribution during optimization.

#### 2.4.3 Principal Component Analysis (PCA) based item reduction

The content was reduced using Principal Component Analysis (PCA). For each subfactor, items with high loadings were identified based on the principal components that explained a substantial portion of the total variance (Moret et al., 2007; Porter et al., 2016). Six items were selected from each subfactor to construct a shortened version of the scale.

#### 2.4.4 Transformer-based item reduction using sentence embeddings

For the transformer-based embedding approach, the model “sentence-t5-xxl” was selected. T5-based sentence embeddings have been reported to achieve over 10% higher correlation scores than BERT-based models on semantic textual similarity (STS) tasks. Furthermore, compared to traditional approaches such as TF-IDF, Word2Vec, and GloVe, T5-based models more accurately capture word order, semantic nuance, and abstract relationships, thereby yielding superior performance in clustering semantically similar items (Ni et al., 2021). Given its expected superiority in embedding quality, *sentence-t5-xxl*—the largest parameterized model within the T5 model series—was chosen for this study. It contains a total of 11 billion parameters and is based on the T5-XXL architecture, which consists of 24 transformer layers, 1,024-dimensional hidden states, 128 attention heads, and 65,536-dimensional feed-forward layers built upon Google's T5 (Text-to-Text Transfer Transformer) framework.

To group semantically similar items, Item texts were first embedded using the sentence-t5-xxl model to capture their semantic relationships in vector form. Since the resulting embeddings were high-dimensional, dimensionality reduction was performed using Uniform Manifold Approximation and Projection (UMAP; McInnes et al., 2018) to enhance the efficiency of clustering and the interpretability of the results. The reduced embeddings were clustered into five groups using K-means, a commonly used clustering algorithm that partitions data into k groups by minimizing the within-cluster variance (Lloyd, 1982). The centroid of each cluster was then computed, and to ensure both representativeness and content diversity, the six items closest to each centroid (based on Cosine distance) were selected. This procedure resulted in a final set of 30 items, preserving the semantic structure of the original item pool.

### 2.5 Validation of the proposed method

#### 2.5.1 Semantic clustering and alignment evaluation

Each IPIP-50 item was embedded into a high-dimensional semantic vector using a transformer-based model. The embedded items were then grouped into five clusters using K-means clustering. To evaluate the alignment between the resulting semantic clusters and the original Big Five factor labels, the Hungarian algorithm was applied to identify the optimal one-to-one mapping (Kuhn, 1955). Classification accuracy was calculated by comparing the mapped cluster assignments with the original factor structure. In addition, to assess cluster separability and cohesion, silhouette scores were computed after reducing the embedding space. The silhouette score is a metric that reflects how closely items are grouped within a cluster and how distinctly each cluster is separated from the others (Rousseeuw, 1987).

#### 2.5.2 Validation against the original scale

To evaluate the extent to which the reduced items retained the psychological properties and structure of the original instrument, we compared the shortened version developed in this study with the full version of the IPIP-50. Specifically, we examined the internal consistency (Cronbach's α) of the original scale and assessed the correlations between scores derived from the original and reduced item sets.

Additionally, these relationships were visualized using correlation matrices to analyze structural consistency, enabling us to evaluate how well the shortened item set preserved the psychometric properties of the original scale. Such visual analyses are commonly employed in scale validation to assess pattern similarity and structural integrity (Eisenbarth et al., 2015). The purpose of this analysis was to determine whether reliability and validity could be maintained despite the reduction in the number of items.

#### 2.5.3 Comparison across item reduction methods

To benchmark the effectiveness of the proposed transformer-based method, we compared it with three alternative approaches: CTT, PCA and a GA. Internal consistency was calculated for each subscale and as an overall average, using the 30-item versions derived from each method.

We then assessed convergent validity by correlating each short form with the full IPIP-50 scale. Finally, predictive performance was evaluated by using each short form to predict item-level scores from the original scale.

Performance was measured using four regression metrics: Mean Absolute Error (MAE), which represents the average absolute difference between predicted and original scores (lower is better); Root Mean Squared Error (RMSE), which emphasizes larger errors by squaring the differences before averaging (lower is better); Coefficient of Determination (*R*^2^), which indicates the proportion of variance in the dependent variable explained by the model (higher is better); and Mean Absolute Percentage Error (MAPE), which expresses prediction accuracy as a percentage of the original scores (lower is better).

Predictions were generated using a deep learning model implemented in PyTorch, consisting of three fully connected layers designed to predict continuous outcomes.

### 2.6 Availability of code

The methods used in this study are documented on Github (https://github.com/sdoublej/teshort/tree/master), where we also provide tools that allow researchers to apply the item reduction procedure themselves. This is intended to enhance the reproducibility and practical utility of the proposed method.

## 3 Results

The results of clustering using the proposed transformer-based embedding method are presented as a confusion matrix in Table 1. Each item from the IPIP-50 was encoded into a semantic vector using a transformer-based model and subsequently grouped via K-means clustering. To evaluate the degree of alignment between the resulting semantic clusters and the original Big Five factor structure, the Hungarian algorithm was applied to find the optimal one-to-one mapping between the semantic clusters and the original factors. The resulting matching yielded an overall accuracy of 96%, and The mean of silhouette score was 0.49.

**Table 1:** Confusion matrix between original Big Five factors and semantic clusters obtained via K-means clustering on transformer-based item embeddings.

**Cluster Label**	**Factor**				
	**AGR**	**CSN**	**EST**	**EXT**	**OPN**
Label: 0				9	
Label: 1			10		
Label: 2		10			
Label: 3					9
Label: 4	10			1	1

Each row represents a semantic cluster label obtained through K-means clustering on transformer-based item embeddings, while each column represents one of the original Big Five personality factors (AGR, Agreeableness; CSN, Conscientiousness; EST, Emotional Stability; EXT, Extraversion; OPN, Openness). For example, of the 10 items originally associated with Agreeableness (AGR), 9 were assigned to semantic cluster Label 1 and 1 item was assigned to Label 3, indicating a high degree of semantic coherence within this cluster.

Table 2 shows the result of item reduction using the proposed method, in which 30 items were selected from five semantic clusters. Items were clearly grouped by their corresponding Big Five subscales within each cluster, indicating strong alignment between semantic structure and the original factor structure. To further assess the quality of this semantic clustering, silhouette scores were examined for each selected item. The average silhouette score was 0.50, suggesting a reasonable degree of cohesion within clusters and separation between them (Rousseeuw, 1987).

**Table 2:** Items selected through transformer-based embedding for short form construction.

**Item_id**	**Item**	**Semantic cluster label**	**Original subscale label**	**Silhouette score**	**Distance**
AGR10	I make people feel at ease.	4	AGR	0.50	0.40
AGR4	I sympathize with others' feelings.	4	AGR	0.46	0.41
AGR5	I am interested in other people's problems.	4	AGR	0.39	0.30
AGR6	I have a soft heart.	4	AGR	0.13	0.34
AGR8	I take time out for others.	4	AGR	0.52	0.44
AGR9	I feel others' emotions.	4	AGR	0.24	0.36
CSN1	I am always prepared.	2	CSN	0.46	0.36
CSN10	I am exacting in my work.	2	CSN	0.67	0.26
CSN2	I keep my belongings organized.	2	CSN	0.61	0.34
CSN4	I keep things tidy.	2	CSN	0.68	0.50
CSN5	I get chores done right away.	2	CSN	0.60	0.42
CSN8	I take responsibility for my duties.	2	CSN	0.58	0.57
EST1	I get stressed out easily.	1	EST	0.70	0.36
EST4	I often feel blue.	1	EST	0.65	0.35
EST5	I am easily disturbed.	1	EST	0.64	0.32
EST6	I get upset easily.	1	EST	0.67	0.31
EST7	I change my mood a lot.	1	EST	0.73	0.44
EST8	I have frequent mood swings.	1	EST	0.71	0.44
EXT1	I am the life of the party.	0	EXT	0.61	0.35
EXT10	I am talkative around strangers.	0	EXT	0.51	0.35
EXT4	I take the lead.	0	EXT	0.35	0.26
EXT6	I have a lot to say.	0	EXT	0.06	0.49
EXT7	I talk to a lot of different people at parties.	0	EXT	0.58	0.45
EXT9	I don't mind being the center of attention.	0	EXT	0.52	0.50
OPN1	I have a rich vocabulary.	3	OPN	0.48	0.71
OPN2	I understand abstract ideas easily.	3	OPN	0.58	0.69
OPN3	I have a vivid imagination.	3	OPN	0.51	0.49
OPN4	I am interested in abstract ideas.	3	OPN	0.31	0.72
OPN6	I have a good imagination.	3	OPN	0.57	0.67
OPN7	I am quick to understand things.	3	OPN	0.13	0.60

Item_id, The item_id indicates the original subscale and item number; Distance, Cosine distance between each item's embedding vector and the centroid of its assigned cluster. A smaller distance indicates closer semantic proximity to the cluster center, representing higher representativeness of that item within the group; Silhouette Score, A measure of how well an item fits within its assigned semantic cluster. Higher values indicate greater cohesion within the cluster and better separation from other clusters.

Table 3 presents the internal consistency and convergent validity of the reduced item set derived using the proposed method. Convergent correlations ranged from 0.95 to 0.98 (*M* = .0.96), and Cronbach's alpha values ranged from 0.73 to 0.85 (*M* = 0.79), all of which indicate acceptable reliability and strong alignment with the original scale. These results support the psychometric adequacy of the proposed transformer-based method in preserving both the factorial structure and measurement quality of the original instrument.

**Table 3:** Convergent correlations of factor scores, total score, and Cronbach's alpha between the transformer-based short form and the original IPIP-50 (*N* = 874,434).

**Factor**	**TE convergent correlations**	**TE Cronbach's alpha**	**Original version Cronbach's alpha**
EXT	0.98	0.85	0.91
EST	0.97	0.82	0.88
AGR	0.95	0.81	0.85
CSN	0.95	0.76	0.83
OPN	0.95	0.73	0.81
Total	0.96	0.79	0.86

TE: Transformer's embedding.

**Figure 1:**
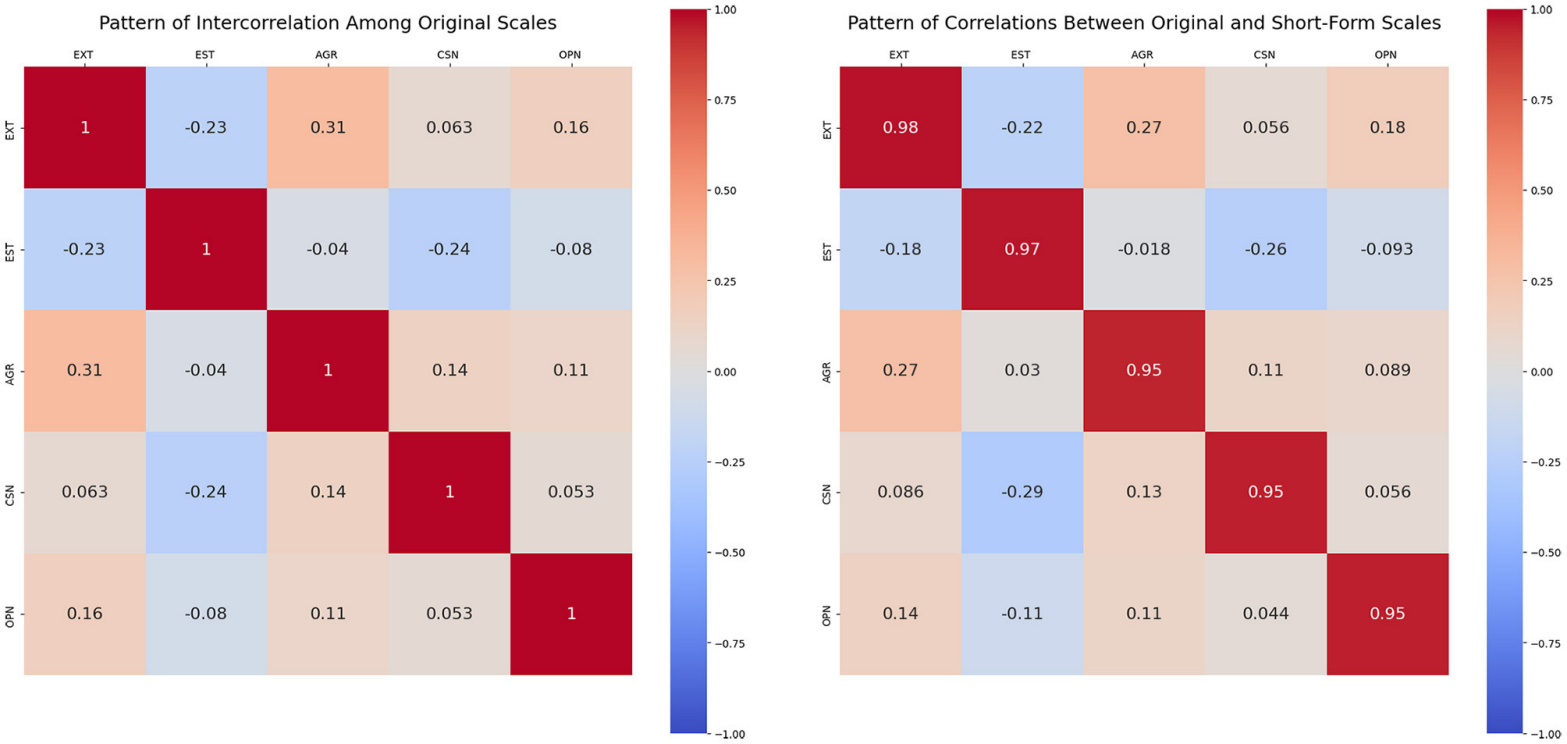
The left panel shows the intercorrelation matrix among the original Big Five factor scores from the IPIP-50 scale. The right panel presents the correlation matrix between the original full-scale scores and the corresponding short-form scores derived using the proposed method. Diagonal values in the right matrix indicate convergent validity between the original and reduced scales. This visual similarity between the two matrices suggests that the proposed short form preserves the structural pattern of interrelationships among the original Big Five traits; AGR, Agreeableness; CSN, Conscientiousness; EST, Emotional Stability; EXT, Extraversion; OPN, Openness.

Figure 1 visualizes the intercorrelations among the original Big Five factors and the correlations between the original and reduced scales. The similarity across panels suggests that the reduced form preserved the factor structure and inter-trait relationships.

Table 4 presents the convergent validity coefficients between each of the shortened item sets and the original IPIP-50 scale, calculated separately for each of the five personality factors, along with the overall mean convergent validity. The CTT-based and PCA-based short forms selected the same set of items, which may be due to the fact that both methods rely on inter-item correlations.

**Table 4:** Convergent validity (r) by factor and mean convergent validity between shortened versions and the original IPIP-50.

**Factor**	**Item reduction method**		
	**CTT/PCA**	**Ga**	**TE**
EXT	0.96	0.98	0.98
EST	0.94	0.97	0.97
AGR	0.93	0.96	0.95
CSN	0.95	0.96	0.95
OPN	0.94	0.96	0.95
Mean	0.94	0.97	0.96

CTT/PCA, dataset reduced using Classical Test Theory–based item selection and Principal Component Analysis; GA, dataset reduced using IRT-informed Genetic Algorithm; TE, dataset reduced using the transformer embedding–based method developed in this study. The CTT- and PCA-based methods selected the same set of items.

The CTT/PCA-based short form yielded an average convergent validity of 0.94, while the GA-based method produced an average of 0.97. The transformer embedding-based method developed in the present study demonstrated an average convergent validity of 0.96. These results indicate that all three item reduction methods maintained strong alignment with the original factor structure, with minor variations in strength across methods.

Table 5 displays the Cronbach's alpha coefficients for the item sets produced by each reduction method—CTT, PCA, GA, and TE proposed in this study. The CTT/PCA-based short form yielded the highest average internal consistency (α = 0.83), followed by the TE-based method (α = 0.79), and the GA-based method (α = 0.78). These findings suggest that while all three methods produced reasonably reliable short forms, CTT/PCA resulted in the highest internal consistency among them.

**Table 5:** Comparison of Cronbach's alpha by factor across shortened versions.

**Factor**	**Item reduction method**			
	**Origin**	**CTT/PCA**	**Ga**	**TE**
EXT	0.91	0.88	0.86	0.85
EST	0.88	0.87	0.83	0.82
AGR	0.85	0.85	0.74	0.81
CSN	0.83	0.81	0.76	0.76
OPN	0.81	0.76	0.71	0.73
Mean	0.86	0.83	0.78	0.79

Table 6 presents the predictive performance of each item reduction method in estimating the original subscale scores. In summary, when predicting under the same conditions, the TE method showed predictive performance that was overall comparable to or better than that of the GA-based and CTT/PCA-based methods in terms of error reduction and explanatory power.

**Table 6:** Prediction performance for original subscale scores using shortened versions from different item reduction methods.

**Metric**	**Item reduction method**		
	**CTT and PCA**	**GA**	**TE**
MAE	2.71	2.70	2.57
RMSE	3.35	3.32	3.17
R^2^	0.81	0.81	0.82
MAPE	8.42%	8.25%	7.97%

MAE, Mean Absolute Error (lower values indicate better fit); RMSE, Root Mean Squared Error (lower is better); R^2^, Coefficient of Determination (higher is better); MAPE, Mean Absolute Percentage Error; (lower is better).

## 4 Discussion

This study aimed to address the limitations of response-dependent short form development methods by proposing a novel item reduction approach using transformer-based semantic embeddings. The proposed method clustered items based on semantic similarity. The Hungarian algorithm was applied to evaluate the alignment between the semantic clusters and the original subfactors, yielding an accuracy of 96%, which reflects a high level of alignment and is considered a strong result for cluster evaluation. Furthermore, the average silhouette score for the selected items was 0.50, suggesting an acceptable level of cohesion within clusters and separation between them. It demonstrates that even without response data, semantic similarity can serve as a reliable basis for organizing psychological items, and suggests the potential for developing short forms that maintain the conceptual integrity of the original scale.

The results demonstrated that applying a transformer-based embedding technique, followed by K-means clustering, effectively grouped items in accordance with their underlying psychological dimensions. These findings suggest that semantic similarity among items was successfully captured through the embedding process, supporting the feasibility of using semantic representations.

Moreover, the short form derived using this method demonstrated high convergent validity with the original scale (correlations > 0.90) and acceptable internal consistency, with Cronbach's alpha coefficients exceeding 0.70 across all factors. Cronbach's alpha values exceeding 0.70 are generally considered acceptable for psychological scales (Taber, 2018).

Visual analysis of item correspondence further confirmed that the structural patterns observed in the full scale were preserved in the reduced form. These findings collectively suggest that the proposed method preserves the core psychometric properties of the original scale, indicating its potential applicability as an efficient and valid tool for psychological assessment.

In addition, when comparing the proposed transformer-based item reduction method with existing approaches—namely, CTT, PCA and GA—CTT/PCA yielded the highest internal consistency (Cronbach's α). This outcome is expected, as CTT selects items based on their correlations with total scores, which tends to inflate internal consistency (Cortina, 1993), And, PCA is also sensitive to inter-item correlations (Jolliffe and Cadima, 2016), which may contribute to the high internal consistency observed in the PCA-based short form.

In terms of convergent validity, however, all three methods yielded similarly strong results:.96 for the transformer-based method,.94 for CTT/PCA, and 0.97 for GA. When comparing predictive performance using key regression metrics (MAE, RMSE, R^2^, and MAPE), the transformer-based method demonstrated performance comparable to that of the GA- and CTT-based methods overall.

Overall, the item reduction method proposed in this study demonstrated competitive performance in terms of reliability, validity, and predictive accuracy when compared with exiting approaches. These findings suggest that transformer-based embeddings may serve as a valid and practical alternative for developing short forms of psychometric instruments.

The contributions of this study are threefold. First, the clustering of numerically embedded items based solely on their semantic content revealed that the factor structure could be recovered without access to response data. This indicates the method's potential for identifying latent dimensions based on item meaning, offering an alternative analytic approach for future exploratory or confirmatory factor analysis.

Second, the study introduces a novel application of transformer-based sentence embeddings—specifically, the sentence-t5-xxl model—in the development of short forms. This highlights the feasibility of using state-of-the-art natural language processing (NLP) techniques to inform item selection in psychological measurement.

Third, the proposed method makes use of semantic similarity between items to offer an alternative way to explore item structure before test administration. This approach could help improve time and cost efficiency in scale development and item refinement.

Despite its strengths, the study has several limitations. First, only one transformer model (sentence-t5-xxl) was employed, and the results may vary depending on the embedding model used. Future research should explore the impact of different embedding architectures on item selection and model performance.

Second, reverse-scored items in the original scale were rephrased into positively worded statements to enable semantic embedding and clustering. While this transformation was necessary for consistent vector representation, it may have altered the original semantic intent or psychometric properties of the items. Prior research (Hommel and Arslan, 2024) suggests that large language models can accurately preserve semantic structure even when negatively worded items are included without rewording. Future studies could therefore explore embedding the original item phrasing without rewording as an alternative. Additionally, to minimize potential researcher bias in the rewording process, objective methods such as automated paraphrasing using the generative capabilities of large language models should be considered.

Third, although the proposed method was compared with CTT, PCA and GA-based approaches, it was not benchmarked against other commonly used item reduction strategies such as Item Response Theory (IRT), factor analysis, or expert judgment. Comparative studies involving a broader range of reduction techniques will be essential to further assess the method's generalizability and relative strengths.

Fourth, while this study primarily focused on internal consistency and basic convergent validity in evaluating the reduced scale, comprehensive scale evaluation should also consider additional indicators, such as model fit, factorial validity, absence of correlated residuals, and especially criterion-related validity, which demonstrates whether the reduced form retains predictive utility. Future research should address these aspects to ensure the robustness of the proposed method.

## Data Availability

The original contributions presented in the study are included in the article/supplementary material, further inquiries can be directed to the corresponding author.
